# Synthesis, transfer, and characterization of core-shell gold-coated magnetic nanoparticles

**DOI:** 10.1016/j.mex.2019.02.006

**Published:** 2019-02-08

**Authors:** McKenzie Smith, Maureen McKeague, Maria C. DeRosa

**Affiliations:** a Chemistry Department, Carleton University, 1125 Colonel By Drive, Ottawa, Ontario, K1S 5B6, Canada; b Department of Pharmacology and Therapeutics, McGill University, 3655 Prom. Sir-William-Osler, Montreal, Quebec, H3G 1Y6, Canada; c Department of Chemistry, McGill University, 801 Sherbrooke Street West, Montreal, Quebec, H3A 0B8, Canada

**Keywords:** Fe_3_O_4_-AuNPs, core-shell gold-coated magnetic nanoparticles, HR-TEM, high-resolution transmission electron microscopy, (HR-TEM/EDS), high-resolution transmission electron microscopy with energy-dispersive X-ray spectroscopy, TMAOH, tetramethylammonium hydroxide, DMAP, 4-dimethyl(amino)pyridine, Solvothermal synthesis of iron oxide nanoparticles with direct gold coating to form core-shell gold-coated magnetic nanoparticles, Phase transfer of core-shell gold-coated magnetic nanoparticles from organic to aqueous using 4-(dimethyl)amino pyridine (DMAP) as a phase transfer agent, Iron oxide, Gold-coated, Core-shell, Core-shell gold-coated magnetic nanoparticles, Magnetic separation, Fe_3_O_4_-AuNPs, 4-Dimethyl(amino)pyridine, DMAP, Nanoparticle phase transfer, Aqueous phase transfer, Solvothermal, Thin gold coating, Nanoparticle, Synthesis

## Abstract

Magnetic separation has gained new popularity as a versatile partitioning method with the recent growth in nanotechnology and related biotechnology applications. In this study, iron oxide magnetic nanoparticles were synthesized via solvothermal methods and directly coated with gold to form core-shell gold-coated magnetic nanoparticles (Fe_3_O_4_-AuNPs). High-resolution transmission electron microscopy with Energy dispersive X-ray spectroscopy results suggests that temperature and reaction time play an important role in the formation of small, monodisperse Fe_3_O_4_-AuNPs. We also demonstrate that increased 4- dimethyl(amino)pyridine (DMAP) concentrations and vigorous stirring were required to successfully transfer Fe_3_O_4_-AuNPs into aqueous solution. The structure and morphology of the synthesized and transferred Fe_3_O_4_-AuNPs was further confirmed by UV–vis absorption spectroscopy and solubility experiments.

• Direct coating of Fe_3_O_4_ with Au: Slowly heating by (10 °C/ min) until 180–190 °C without exceeding this reaction temperature and increasing the reaction time to 3 h from 1.5 h
• High yield transfer of Fe_3_O_4_-AuNPs was achieved using 4- dimethyl(amino)pyridine (DMAP) as phase transfer catalyst

## Graphical abstract

**Figure:**
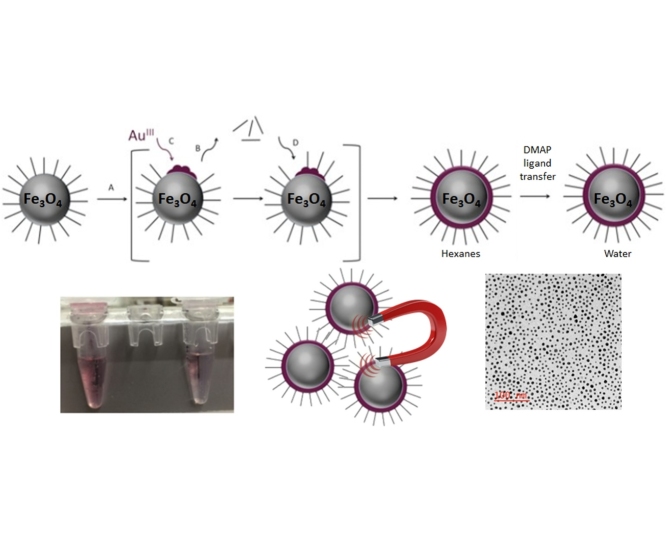

**Specifications Table**

**Table:** 

Subject area	• *Chemistry*
More specific subject area	*Core-shell nanoparticle synthesis and phase transfer*
Method name	• *Solvothermal synthesis of iron oxide nanoparticles with direct gold coating to form core-shell gold-coated magnetic nanoparticles,* • *Phase transfer of core-shell gold-coated magnetic nanoparticles from organic to aqueous using 4-(dimethyl)amino pyridine (DMAP) as a phase transfer agent*
Name and reference of original method	• *Monodispersed Core-Shell Fe_3_O_4_-Au Nanoparticles* • *Synthesis of core-shell gold coated magnetic nanoparticles and their interaction with thiolated DNA* • *Spontaneous phase transfer of nanoparticulate metals from organic to aqueous media*
Resource availability	• *10.1021/jp0543429* • *10.1039/c0nr00621a* • *10.1002/1521-3773*

## Method details

### Overview

Nanoparticles provide an increased surface area to volume ratio and unique physicochemical properties that are useful in a wide range of applications [[1], [2], [3]]. Core-shell nanoparticles are a class of nanoparticles consisting of an inner core nanoparticle that is coated with a different material as an outer shell. Various core-shell nanoparticles ranging in size, shape, surface coverage and morphology have been synthesized and reported. However, the most common core-shell nanoparticle shape is the concentric spherical which consists of a complete covering of a spherical nanoparticle with another material. This system is advantageous because the chemical or physical properties of a nanoparticle surface can be altered without losing the properties provided by the core material [4]. As a result of the increased functionality within a single system, core-shell nanoparticles have great potential in a wide range of applications including biomedical [5,6] and pharmaceutical [7] applications, catalysis [8], electronics [9], and optics [10].

While iron oxide nanoparticles (Fe_3_O_4_ NPs) have been successfully used as platforms for rapid and versatile partitioning methods, they are limited in their widespread application. Fe_3_O_4_ NPs have a large surface area to volume ratio with a low surface charge at neutral pH which typically leads to aggregation. In addition, Fe_3_O_4_ NPs have low electrical conductivity and poor optical properties [[11], [12], [13], [14], [15]]. One solution is to coat magnetic nanoparticles with another material, such as gold. The gold coating has the additional benefit of enhancing the surface biocompatibility [16,17], bioaffinity [18], optical properties [19], chemical stability [20] and conductivity [21] of the nanoparticle with that of gold, while maintaining the magnetic properties of iron oxide. Therefore, methods for the efficient preparation of Fe_3_O_4_-AuNPs are of interest in order to facilitate their use in a wide range of applications, including bioseparation [16], electrochemical sensors [22], targeted delivery [23] and bioimaging [24]. In particular, these bionanotechnology applications would require the nanoparticle to be dispersed in water.

Synthesis methods play an important role in the successful production of small, mono-disperse core-shell nanoparticles. Furthermore, for each application, the size, coating, and composition must be tunable, yet reliable. Therefore, many synthesis methods have been developed in an attempt to produce nano-sized core-shell particles. Fe_3_O_4_-AuNPs have been synthesized using hydroxylamine seeding [18], reverse micelles [25], Y-ray radiation [26], laser ablation [27], sonochemical [28] and wet chemical reactions [29], layer-by-layer electrostatic deposition [30], and photochemical reduction [31]. However, many of these synthesis methods either require costly, specialized equipment or are known to produce larger nanoparticles (>60 nm). Notably, Fe_3_O_4_-AuNPs have been formed through a sequential synthesis process where iron oxide nanoparticles were synthesized and, subsequently, directly coated with gold through nucleation of gold on the surface. This simple synthetic method has been reported to easily produce small core-shell gold-coated magnetic nanoparticles (Illustration 1) [32]. However, this method served as the foundation for this work as certain reaction conditions and modifications described herein were found to be essential to achieve the monodispersity and quality required for our application [33].

**Illustration 1:**
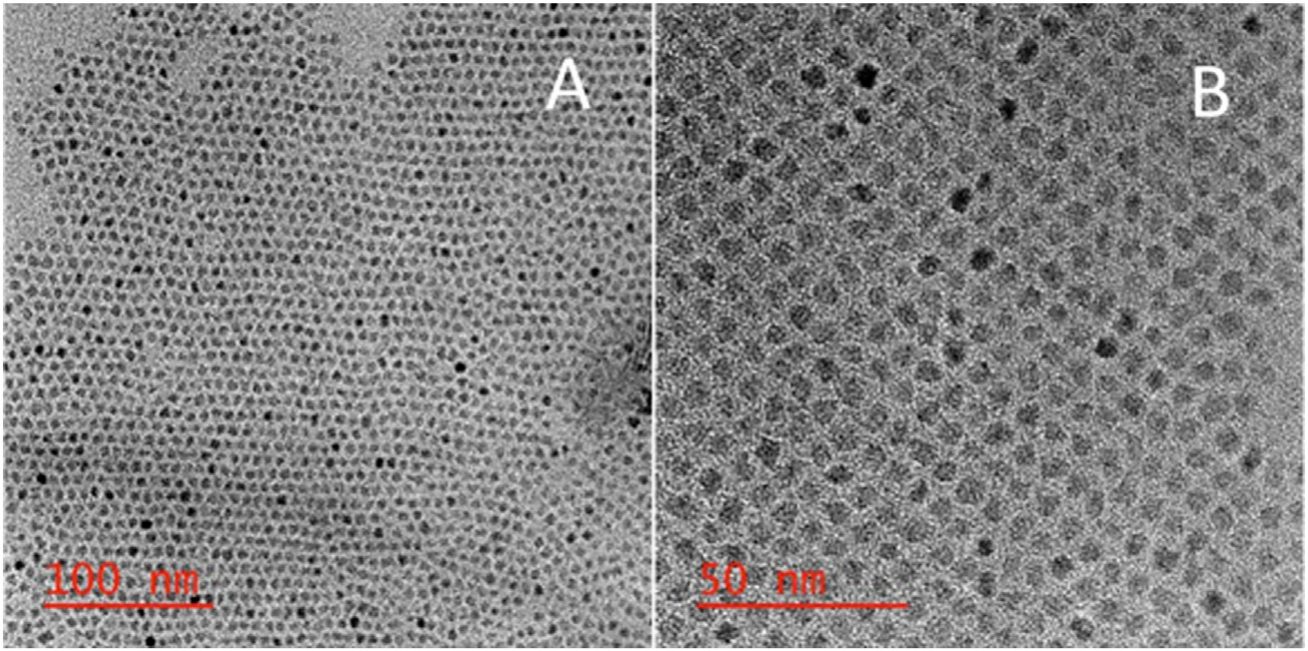
Illustration of the coating process; (A) Au(CH_3_COO-)_3_ is reduced by 1,2- hexadecanediol in the presence of oleic acid and oleylamine at a temperature of 180–190 °C, (B) thermally active partial desorption of the capping layer occurs, (C) the reduction of Au(CH_3_COO-)_3_ results in deposition of Au on the surface of the exposed surface of the magnetic nanoparticles, and (D) the re-encapsulation of the Au shell with oleylamine and oleic acid.

Iron oxide (Fe_3_O_4_) nanoparticles are commonly synthesized through solvothermal methods. Reducing Fe(acac)_3_ with 1,2- hexadecanediol in the presence of oleic acid and oleylamine successfully produces Fe_3_O_4_ seeds. This synthesis method is advantageous as it a simple procedure capable of producing small, monodisperse and stable iron oxide nanoparticles [34]. In addition, using oleylamine and oleic acid as stabilizing agents allows the previously synthesized iron oxide nanoparticles to be easily and directly coated with gold through a thermally active process [35].

As for most synthetic procedures, many factors must be controlled for the successful synthesis of core-shell nanoparticles. The rate at which the reaction temperature is increased, the reaction time and the precise control of the reaction temperature not only improved the synthesis of the iron oxide nanoparticle core, but also improved the stability, uniform coating, and composition of the final core- shell nanoparticle product. Small monodisperse gold-coated magnetic nanoparticles were required for our aqueous separation applications. Following procedures described by Robinson et al., it was found that we could not achieve a monodisperse iron oxide sample [32]. In addition, we found that this procedure was unable to successfully produce uniform coverage of the particles, resulting in mixtures of uncoated and coated particles. Thus, by following Wang et al. and further optimizing the reaction conditions, we were able to synthesize small, monodisperse Fe_3_O_4_-AuNPs with a consistent, thin gold coating [33]. In this work, core-shell gold-coated magnetic nanoparticles were synthesized with various reaction parameters to determine the importance of reaction time and temperature for this synthetic process. To characterize the final Fe_3_O_4_-AuNPs product, high-resolution transmission electron microscopy (HRTEM) with Energy dispersive spectroscopy (EDS), and UV–vis absorption spectroscopy were employed. Finally, we needed to transfer the nanoparticles into water, requiring a phase transfer step. Phase transfer of the synthesized Fe_3_O_4_-AuNPs with tetramethylammonium hydroxide (TMAOH), a capping agent used by Robinson et al. and Wang et al., led to aggregation and reduced yield [32,33].

Therefore, we attempted to transfer the synthesized core-shell nanoparticles from hexanes to water

using 4-dimethyl(amino)pyridine (DMAP) as a phase transfer catalyst. Previously, Gittins and Caruso used DMAP to transfer tetraoctylammonium bromide (TOAB) capped gold nanoparticles from toluene to water through a proposed ligand exchange mechanism [36]. In this work, DMAP was used to transfer Fe_3_O_4_-AuNPs from hexanes to water. It is proposed that DMAP displaces oleylamine and oleic acid to transfer the nanoparticles across the solvent layer, providing solubility in water for bionanotechnology applications. The transferred DMAP-capped Fe_3_O_4_-AuNPs were characterized with HRTEM/EDS and through solubility experiments (Illustration 2).

**Illustration 2:**
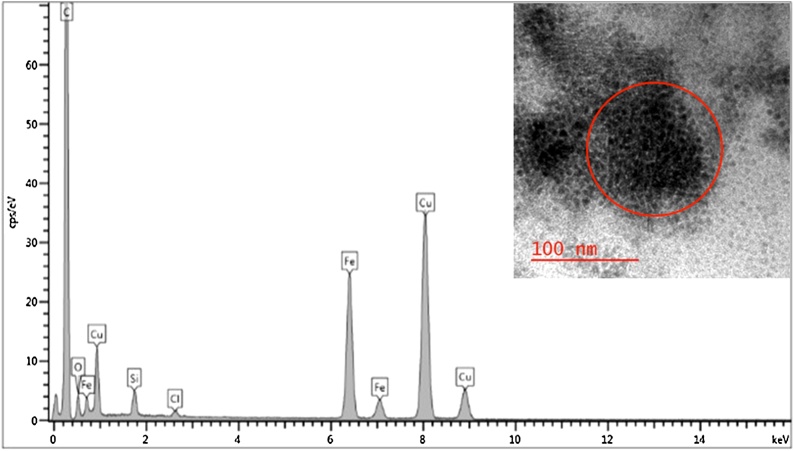
Illustration of the proposed ligand exchange phase transfer of AuNPs from hexanes (capped with oleylamine and oleic acid) into aqueous solution using DMAP.

## Experimental

### Materials

Iron (III) acetylacetonate (Fe(acac)_3_), 99%, oleylamine (70%), oleic acid (99%), phenyl ether (99%), anhydrous sodium citrate (99%), 4-dimethyl(amino)pyridine (99%) and other solvents (hexanes, toluene and absolute ethanol) were purchased from Sigma-Aldrich (Oakville, ON, Canada). 1,2-Hexadecanediol was purchased from VWR (Mississauga, ON, Canada). Gold (III) acetate (Au(ac)_3_) was purchased from Alpha Aesar (Haverhill, Massachusetts, USA). 300 mesh × 83 μm pitch copper TEM grids were purchased from Ted Pella (Redding, CA, USA). All glassware used for the Fe_3_O_4_-AuNP synthesis was washed with aqua regia (3:1 mixture concentrated HCl/HNO_3_) and rinsed thoroughly with deionized water. The glassware was then rinsed with acetone before being dried in an overnight or until used for synthesis.

Unless otherwise specified, all synthetic procedures were performed using standard Schlenk techniques under an argon (5.0, 99.999%) atmosphere.

### Synthesis of Fe_3_O_4_ nanoparticles

0.71 g of Fe(acac)_3_ was dissolved in 20 mL of phenyl ether with 2 mL of oleic acid and 2 mL of oleylamine (end volume of 24 mL) with vigorous stirring. Once dissolved, 2.58 g of 1, 2-hexadecanediol was added. A water cooled condensing column was fitted to the flask. The solution was slowly heated to 210 °C as to ensure the temperature did not exceed 210 °C, and kept at reflux for 2 h at this temperature. After 2 h, the bright red suspension appeared dark brown in colour, and was allowed to cool to room temperature under argon overnight.

### Direct coating of Fe_3_O_4_ nanoparticles to form Fe_3_O_4_-AuNPs

10 mL of previously prepared Fe_3_O_4_ in phenyl ether (above reaction product) was added to 30 mL of phenyl ether with vigorous stirring. To this solution, 0.83 g of Au(ac)_3_, 3.1 g of 1,2-hexadecanediol, 0.5 mL of oleic acid and 3 mL of oleylamine were added quickly. This suspension was heated slowly (10 °C/ min) until 180–190 °C and maintained at this temperature under reflux for 3 h. The solution was allowed to cool to room temperature under argon overnight. 5 mL of the solution was transferred to a 125 mL Erlenmeyer flask and 15 mL of ethanol was added to it. The solution was agitated gently resulting in a visible aggregation of the nanoparticles and a colour change from dark purple to dark blue/black. The flask was then placed on a magnet for 5–10 min to magnetically separate the particles from the supernatant solution. The supernatant solution was decanted as waste and the precipitated nanoparticles were washed three times with 15 mL of absolute ethanol. The nanoparticles were then redispersed in a solution of 10 mL of hexanes, 0.25 mL of oleic acid and 0.25 mL of oleylamine. This procedure was repeated multiple times to obtain purified Fe_3_O_4_-AuNPs in hexanes. Solutions appeared dark red-purple in colour, and were stored in glass covered by foil at room temperature.

### Transfer of Fe_3_O_4_-AuNPs with 4-dimethyl(amino)pyridine

The following methods were not performed under Argon. 0.5 M DMAP solution was prepared by adding 0.68 g of DMAP into 1 mL of Milli-Q 18.2 Ω water. 1 mL of Fe_3_O_4_-AuNPs was added to a 1 mL aliquot of 0.5 M aqueous DMAP solution in a glass vial. Two phases were observed, a dark purple hexane layer (top) and a clear aqueous layer (bottom). The phases were thoroughly mixed with vigorous stirring (via a magnetic stir rod and plate) for 1 h. At this time, the top hexane layer appeared light purple to clear and the bottom aqueous layer appeared dark purple suggesting that the nanoparticles were successfully transferred into aqueous solution. The bottom layer was transferred with a Pasteur pipette to a new glass vial and purified by washing the nanoparticles with a 0.5 M DMAP solution via magnetic separation.

### Characterization of synthesized Fe_3_O_4_-AuNPs

The UV–vis absorption characterization of the Fe_3_O_4_-AuNPs was performed using a Cary 300 Bio UV–vis spectrophotometer (Varian, Santa Clara CA). Fe_3_O_4_-AuNPs were prepared as described and analyzed at each step during synthesis. Transmission electron micrographs were taken with a FEI Technai G2 F20 TEM at the Carleton University Nano-imaging Facility, with a field emission source at a voltage of 200 kV using Gatan Microscopy Suite 2 V. All images were taken on dry 300 mesh × 83 μm pitch carbon coated copper TEM grids at room temperature. Grids were prepared by placing 4 μL of Fe_3_O_4_-AuNP (in various solvents) on a TEM grid. The TEM grids were allowed to dry for 4–24 h depending on the solvent. Images were taken at 1–2 μm, 100–200 nm and 5–10 nm for each grid. EDS of each Fe_3_O_4_-AuNPs sample was taken at a 20° take off angle with an Oxford X-ma × 80 mm EDS detector using Aztec software. Transmission electron micrograph images were analyzed for nanoparticle size distribution using ImageJ software. The scale of the image was reset from metres to pixels.

Bandpass filter and threshold were used to improve the resolution of the image for analysis. The area of each nanoparticle was determined with the nanoparticle analysis function. A histogram was assembled for the frequency of each nanoparticle diameter, assuming perfect sphericity. The average nanoparticle diameter with standard deviation for a number of nanoparticles was calculated.

## Results and discussion

### Iron oxide nanoparticles synthesis

**Fig. 1:**
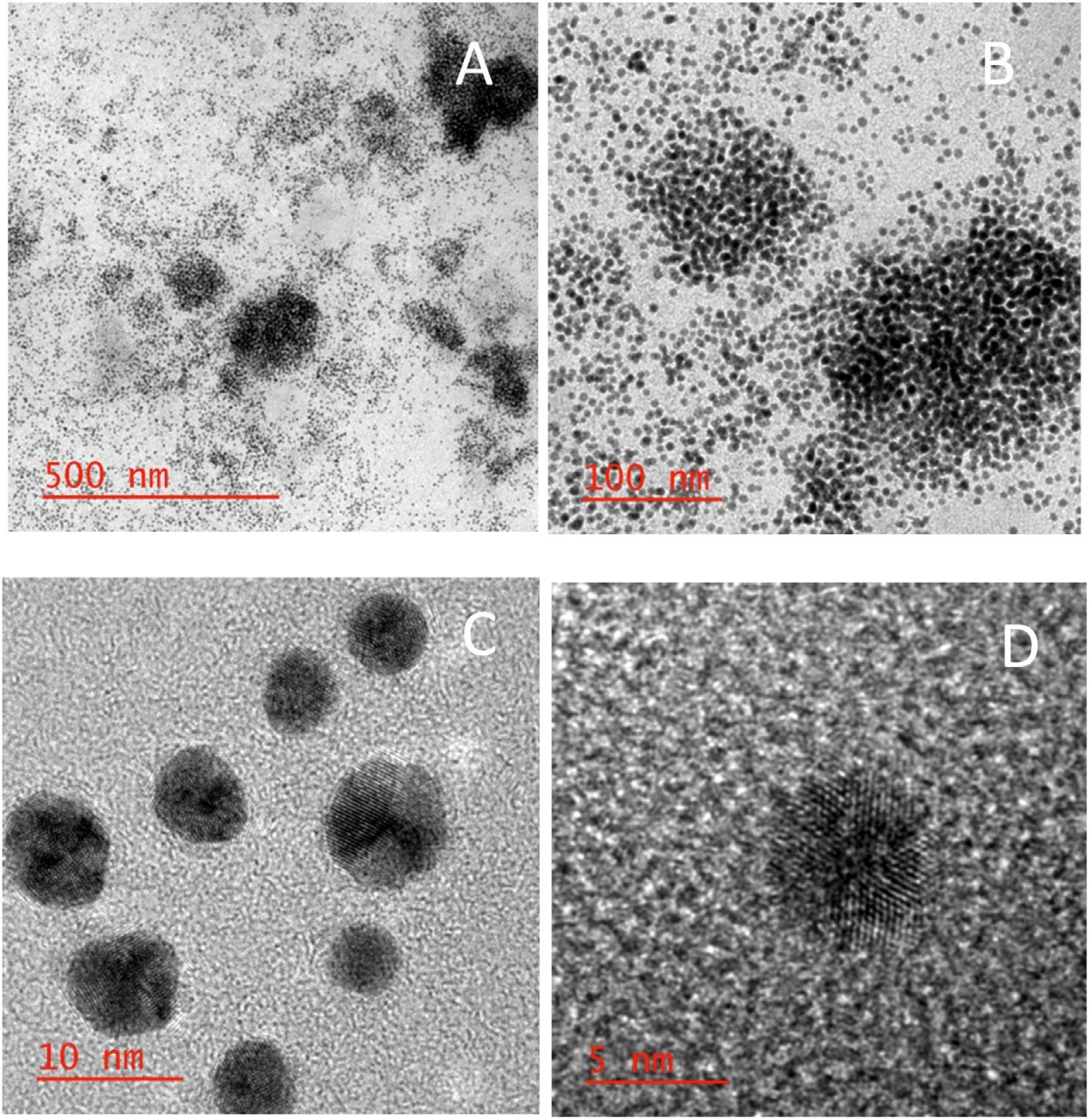
HRTEM images of small, monodispersed Fe_3_O_4_ NPs in diphenyl ether with 100 and 50 nm scale bars.

**Fig. 2:**
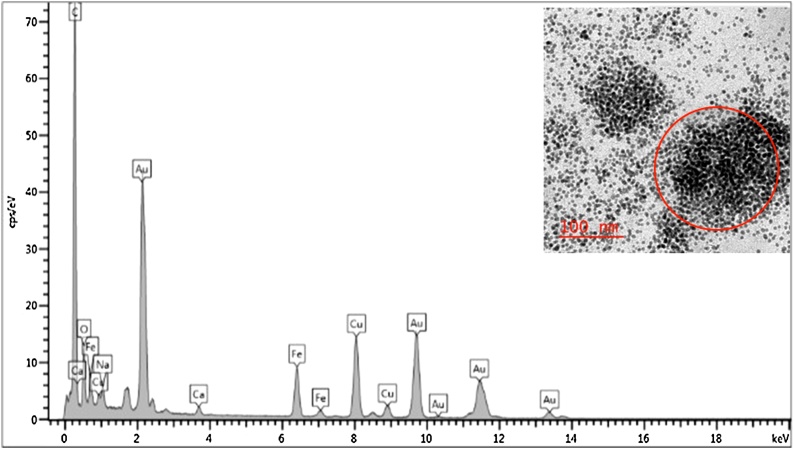
HRTEM image with corresponding EDS spectrum of synthesized Fe_3_O_4_ NPs in diphenyl ether. A strong EDS peak is observed for carbon due to carbon-coated TEM grid. Peaks for both Fe and O depict the presence of iron oxide nanoparticles.

Robinson et al. and, originally, Sun and Zeng report the synthesis of size-controlled magnetite nanoparticles through a solvothermal method where Fe(acac)_3_ is reduced by 1,2-hexadecanediol in the presence of two capping agents (oleylamine and oleic acid) [31,33]. Fe(acac)_3_ was mixed with 1,2- hexadecandiol, oleylamine, and oleic acid in phenyl ether under nitrogen and heated to reflux [31]. However, when the solution was heated to the boiling point of phenyl ether (258 °C) for 2 h, the size distribution of the synthesized magnetite nanoparticles was quite extensive (Fig. S1.) Alternatively, when the temperature of the solution was heated to 210 °C, as was suggested by Wang et al, the size distribution of the synthesized magnetite nanoparticles was narrowed producing more mono-disperse Fe_3_O_4_ NPs [32]. HRTEM characterization displays the size-controlled synthesis of small, mono-disperse Fe_3_O_4_ NPs (Fig. 1) with an average diameter of 5.96 nm (σ = 0.23 nm, n = 7) (Fig. S9). Furthermore, HRTEM/EDS confirms that the nanoparticles are composed of iron oxide (Fig. 2).

### Gold coating to form Fe_3_O_4_-AuNPs

Following Robinson et al., the synthesized Fe_3_O_4_ were cooled to room temperature and used without any further separation [32]. Fe_3_O_4_ NPs were coated with gold by reducing gold acetate with 1,2- hexadecanediol in the presence of the previous formed Fe_3_O_4_ NPs, oleylamine and oleic acid in phenyl ether. Under inert atmosphere, the solution was heated to 180–190 °C and maintained at this temperature with vigorous stirring for 1.5 h. Unexpectedly, the HRTEM images suggested that the complete coating of the Fe_3_O_4_ NPs with gold was unsuccessful, and instead the synthesis produced two distinct sizes of nanoparticles (Fig. S3). HRTEM with EDS confirmed that the smaller particles were uncoated iron oxide, and the larger particles were AuNPs or Fe_3_O_4_ NPs coated with gold (Fig. S4).

It is suggested that temperature plays an important role in the partial desorption of the Fe_3_O_4_ NP capping agents and is required for the reduced gold acetate to directly coat the exposed Fe_3_O_4_ NPs. The partial desorption of the Fe_3_O_4_ NPs capping agents occurs at a specific temperature as it is a thermally active process. Therefore, if the temperature is increased suddenly, AuNPs may begin to form separately since the Fe_3_O_4_ NP surface is unavailable as a nucleation site. Wang et al. report a precise, incremental increase in temperature by 10 °C/min until a temperature of 180–190 °C was achieved [33]. Again, this temperature was maintained for 1.5 h. In addition to incorporating this incremental increase in reaction temperature, the solution was maintained at this temperature for a longer period of time (3 h). These changes were found to greatly improve the final Fe_3_O_4_-AuNP product.

**Fig. 3:**
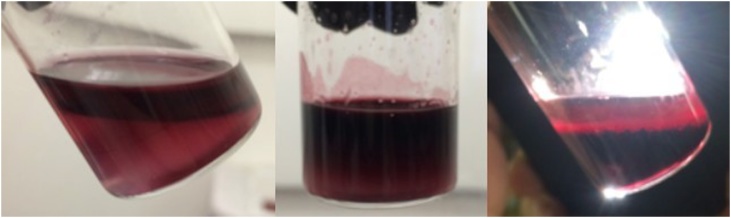
HRTEM images of synthesized Fe_3_O_4_-AuNPs with 500, 100, 10 and 5 nm scale bars.

**Fig. 4:**
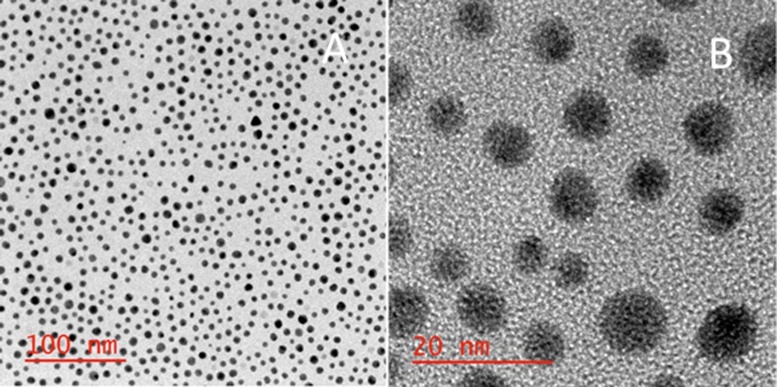
HRTEM image with corresponding EDS spectrum of synthesized Fe_3_O_4_-AuNPs in hexanes prior to phase transfer. A strong EDS peak for carbon is observed due to the use of carbon-coated TEM grid. Peaks for both Fe and Au indicate the presence of Fe_3_O_4_-AuNPs.

The synthesized Fe_3_O_4_-AuNPs were characterized by HRTEM/EDS and UV–vis absorption spectroscopy. Fig. 3 displays the small, mono-disperse Fe_3_O_4_-AuNPs and HRTEM/EDS confirms the presence of both iron oxide and gold (Fig. 4). The {111} lattice spacing on the surface of the synthesized core-shell nanoparticles was found to be approximately 0.243 Å which is characteristic of gold (Fig. S8).

While no uncoated Fe_3_O_4_ NPs were observed on the TEM grids, it was important to ensure that the particles imaged were of a core-shell structure with the expected composition, not just AuNPs without a magnetic core. First, it was noted that the average size of the imaged nanoparticles increased from 5.96 nm (σ = 0.23 nm, n = 7) to 6.53 nm (σ = 1.18 nm, n = 71), which is attributed to the addition of a gold shell (Figs. S5 and S6). Furthermore, the samples imaged were separated by magnetic separation prior to deposition onto the TEM grids, which should preclude the deposition of any AuNPs lacking a magnetic core. This was further confirmed by imaging a mixture of separately synthesized AuNPs and Fe_3_O_4_ nanoparticles before and after magnetic separation and comparing these TEM/EDS results with the TEM/EDS results of our synthesized Fe_3_O_4_-AuNP product (Figs. S11 and S12).

Therefore, it is concluded that using stable, mono-disperse Fe_3_O_4_ NPs, increasing the reaction temperature at precisely 10 °C/minute and heating the solution for 3 h instead of 1.5 h greatly influenced the success of the Fe_3_O_4_-AuNP synthesis.

### DMAP transferred Fe_3_O_4_-AuNPs

Aqueous nanoparticle dispersibility is essential for many applications, in particular for biological purposes. Since the aforementioned synthesis is conducted in organic solvent (phenyl ether), the capping agents (oleylamine and oleic acid) allow the nanoparticles to be dispersed in non-polar solvents such as hexanes. Therefore, ligand exchange must be accomplished to achieve dispersibility in water.

Tetramethylammonium hydroxide (TMAOH) has been reported to replace capping agents on the surface of synthesized Fe_3_O_4_-AuNPs. In doing so, the capping agents present during synthesis can be replaced through a ligand exchange with a water-soluble capping agent such as citrate. Robinson et al. and Tintore et al. reported that the original oleylamine /oleic acid capping agents could be replaced with citrate when the synthesized Fe_3_O_4_-AuNPs were first washed with 1 M TMAOH [32,37].

**Fig. 5:**
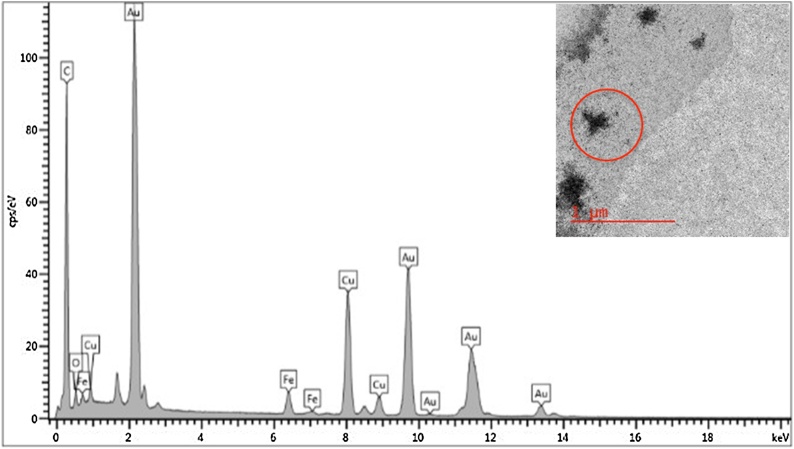
Images of synthesized Fe_3_O_4_-AuNPs before (left), during (middle) and after (right) DMAP ligand exchange phase transfer.

**Fig. 6:**
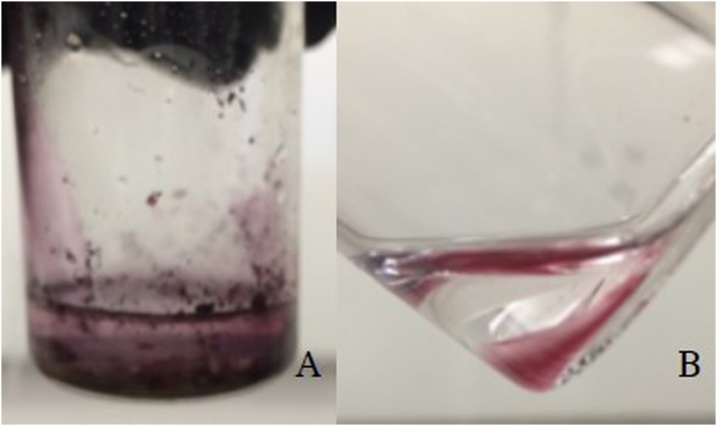
HRTEM image of transferred DMAP-capped Fe_3_O_4_-AuNPs in water with 100 and 20 nm scale bar.

**Fig. 7:**
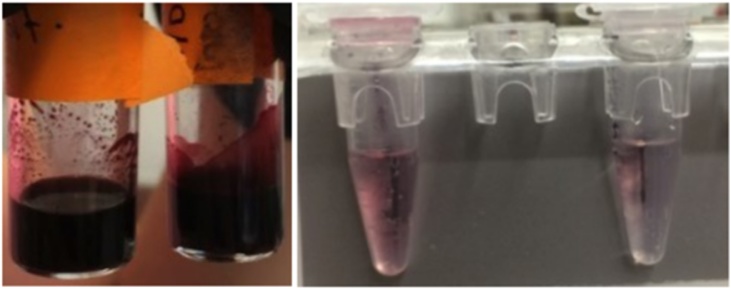
HRTEM image with corresponding EDS spectrum of transferred DMAP- capped Fe_3_O_4_-AuNPs in aqueous solution. Peaks for both Fe and Au depict the presence of Fe_3_O_4_-AuNPs.

When the phase transfer was attempted, although it was successful, the yield was insufficient for our future separation-based applications (Fig. S10). Visible by HRTEM, the transferred citrate capped Fe_3_O_4_-AuNPs were so dilute as to be colorless solutions. This may not be concerning for applications such as bio-imaging, however this is problematic for applications that require a visible indication that the Fe_3_O_4_-AuNPs are being separated from solution via magnetic separation. Therefore, reducing aggregation and loss of product during transfer was of interest. Previously, Gittins and Caruso reported transfer of AuNPs from organic solvent to aqueous solution using 4-dimethyl(amino)pyridine (DMAP) as a phase transfer catalyst [36]. It was reported that tetraoctylammonium bromide (TOAB) was replaced by DMAP on the AuNP surface resulting in a spontaneous transfer of the AuNPs from toluene to water within 1 h. Adsorption of the endocyclic nitrogen of DMAP, is proposed to displace of the primary amine of oleylamine on the AuNP surface through a ligand exchange. Compared to the primary amine of oleylamine, DMAP creates a stronger bond with the gold atoms on the nanoparticle surface. This is attributed to the charge localization of the DMAP conjugate acid, which raises the energy of the lone pair on the nitrogen atom [37]. The position of the ligand’s HOMO and LUMO orbitals in relation to the Fermi level of Au determines the strength of the Au-ligand interaction. This is further explained by the Hard-Soft Acid-Base theory [38]. When metals (including Au) are in a 0 oxidation state, they are considered a soft acid [39,40]. HSAB classifies DMAP as a borderline to soft base, while the primary amine of oleylamine is a relatively hard base.Therefore, a soft base, like DMAP, interacts more strongly with the gold nanoparticle surface in comparison and will likely replace oleylamine via phase transfer. In our study, the Fe_3_O_4_-AuNPs were capped with oleylamine/oleic acid and were dispersed in hexanes. These differences were noted and accounted for by increasing the concentration of DMAP (0.1 M–0.5 M) and vigorously stirring the solution during transfer. The synthesized Fe_3_O_4_- AuNPs were successfully transferred into water through a proposed ligand exchange mechanism. It is suggested that, according to Hard-Soft Acid-Base (HSAB) theory, the endocyclic nitrogen of DMAP interacts more strongly with the gold nanoparticle surface in comparison to oleylamine/oleic acid [36,[40], [41], [42], [43], [44]]. Fig. 5 displays the location of the Fe_3_O_4_-AuNPs over time during transfer. Before transfer, the Fe_3_O_4_-AuNPs capped with oleylamine/oleic acid are soluble in hexanes (top layer). Adding a 0.5 M solution of DMAP in water results in the Fe_3_O_4_-AuNPs spontaneously transferring across the phase boundary into the water layer. After 1 h with vigorous stirring, it was found that most of the Fe_3_O_4_- AuNPs were transferred and soluble in water. The transferred DMAP-capped Fe_3_O_4_-AuNPs were characterized by HRTEM/EDS and UV–vis absorption spectroscopy. Although there still remained a notable decrease in yield, transfer of the Fe_3_O_4_-AuNPs with DMAP was found to be successful. Fig. 6 displays HRTEM images of mono-disperse Fe_3_O_4_-AuNPs. HRTEM/EDS confirms the expected core-shell gold-coated magnetic nanoparticle morphology (Fig. 7). The size distribution is estimated to be consistent with the oleylamine/oleic acid capped Fe_3_O_4_-AuNPs in hexanes, with the DMAP capped Fe_3_O_4_- AuNPs having an approximate average diameter of 6.45 nm (σ = 1.07 nm, n = 73) (Fig. S7). Furthermore, although the concentration decreases, UV–vis absorption spectroscopy displays an absorption peak at approximately 535 nm corresponding to mono-disperse Fe_3_O_4_-AuNPs (Fig. 9).

### Dispersion of Fe_3_O_4_-AuNPs with/without DMAP ligand exchange phase transfer

**Fig. 8:**
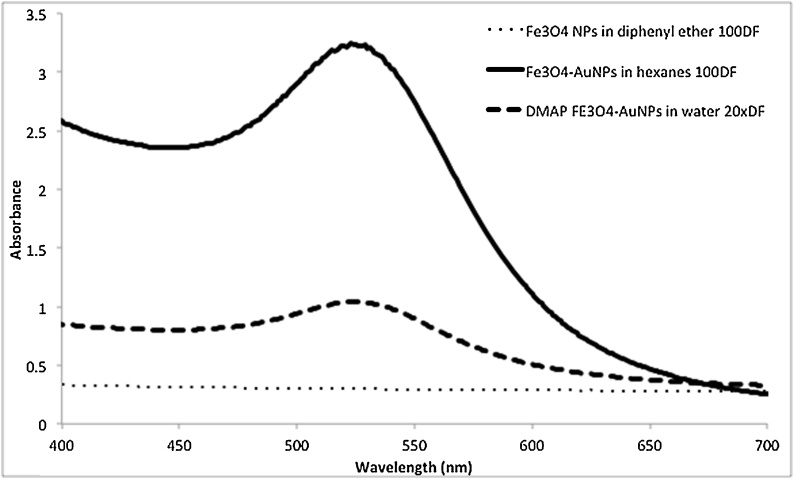
Images of oleylamine/oleic acid-capped Fe_3_O_4_-AuNPs in water (A), DMAP-capped Fe_3_O_4_-AuNPs in hexanes (B) and HRTEM image of image A with a 2 μm scale bar.

To confirm that the original capping agents (oleylamine and oleic acid) of the synthesized Fe_3_O_4_- AuNPs were successfully replaced by DMAP, dispersibility of the original and ligand-exchanged Fe_3_O_4_- AuNPs was tested. Fig. 8A. displays oleylamine/oleic acid capped Fe_3_O_4_-AuNPs in water without replacing the capping agents with DMAP. The Fe_3_O_4_-AuNPs are not soluble in water when capped with oleylamine and oleic acid. This is confirmed by HRTEM characterization which displays extensive, irreversible solvent induced aggregation (Fig. 8). Similarly, the DMAP-capped Fe_3_O_4_-AuNPs were added to a solution of hexanes. Fig. 8B. displays the lack of colour in the top layer (hexanes) confirming the absence of the nanoparticles in that phase and suggesting that the ligands on the Fe_3_O_4_-AuNPs were replaced with water soluble DMAP.

### Magnetic separation DMAP coated Fe_3_O_4_-AuNPs

**Fig. 9:**
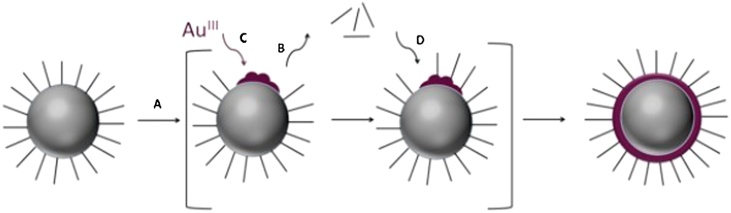
Images of DMAP capped Fe_3_O_4_-AuNPs after precipitation with DMAP in hexanes without (A1) and with (A2) magnetic separation. and DMAP capped Fe_3_O_4_-AuNPs after ligand exchange transfer with DMAP in aqueous solution without magnetic separation (B1) and with magnetic separation (B2).

When placed on the magnet, the DMAP transferred sample has clearer separation than the nanoparticles transferred with DMAP precipitation (Fig. 9). The precipitated Fe_3_O_4_ –AuNPs could be precipitated and washed with magnetic separation, however this would only result in a decreased concentration of nanoparticles in solution. Therefore, ligand exchange of Fe_3_O_4_-AuNPs with DMAP is recommended for transfer and magnetic separation (Fig. 9B).

### UV–vis absorption spectroscopy

**Fig. 10:**
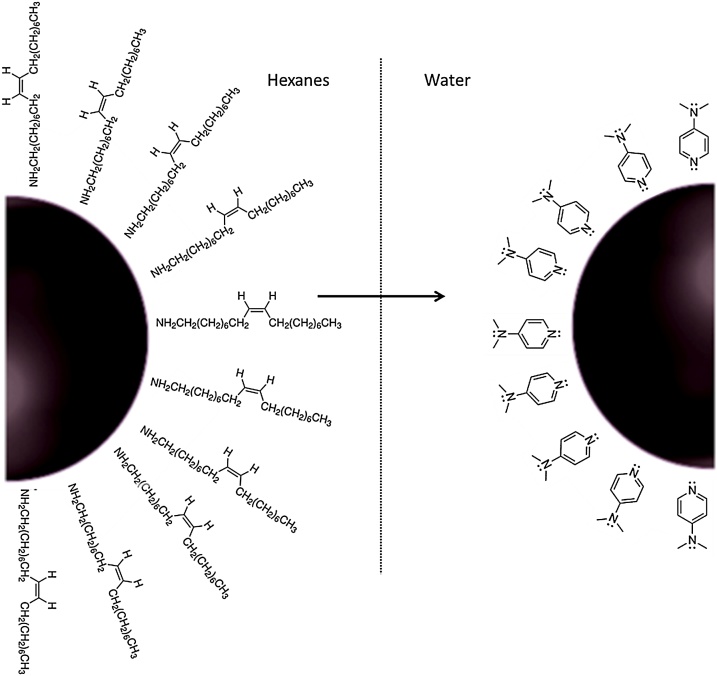
UV–vis spectra of Fe_3_O_4_ NPs (100 times dilution factor of stock), Fe_3_O_4_-AuNPs (100 times dilution factor of stock) and transferred Fe_3_O_4_-AuNPs (20 times dilution factor of stock).

The unique physical and optical properties of AuNPs, specifically in comparison to magnetite nanoparticles, permits the use of UV–vis absorption spectroscopy to further confirm the presence of a gold shell. UV–vis absorption spectrum of the core- shell nanoparticles can be insightful. Compared to AuNPs of the same size, a core Fe_3_O_4_ results in a slight red shift in the absorption, which can support other characterization methods. A thinner coating of Au on the Fe3O4 nanoparticles results in a spectrum that experiences a larger red-shift from that of AuNPs (˜525 nm). This shift results in absorption of approximately 530–540 nm for core- shell Fe_3_O_4_-AuNPs depending on the thickness of the Au shell [3]. In addition, the concentration of the Fe_3_O_4_-AuNP sample before and after phase transfer can be estimated to evaluate its efficiency. Fig. 10. displays the absorption spectra for Fe_3_O_4_ NPs, Fe_3_O_4_- AuNPs capped with oleylamine/oleic acid in hexanes and Fe_3_O_4_-AuNPs capped with DMAP in water. The localized surface plasmon resonance (LSPR) of AuNPs results in an absorption peak between approximately 520–550 nm depending on the diameter of the AuNP. The absorption peak at approximately 535 nm corresponds to the Fe_3_O_4_-AuNPs sample. In comparison to AuNPs, the absorption peak of the Fe_3_O_4_-AuNPs of the same size is red-shifted due to the core-shell structure [35,45]. It is noted that the concentration of DMAP capped Fe_3_O_4_-AuNPs is much lower than the synthesized Fe_3_O_4_-AuNPs in hexanes. However, the DMAP capped Fe_3_O_4_-AuNPs were less aggregated during transfer and, therefore, more concentrated in water than the TMAOH transferred citrate capped Fe_3_O_4_-AuNPs sample (Fig. S10).

## Conclusions

This study highlights the importance of reaction temperature for the synthesis of small, mono- disperse magnetite nanoparticles and successful, direct coating of these nanoparticles with gold to form Fe_3_O_4_-AuNPs. Our work displays how a slight deviation in reaction temperature during the synthesis of Fe_3_O_4_ NPs results in a large size distribution and lack of core-shell formation in the subsequently prepared Fe_3_O_4_-AuNPs. Notably, we found that failing to precisely increase the reaction temperature led to the formation of separate AuNPs, or an inconsistent gold shell, in the presence of Fe_3_O_4_ NPs. In addition, a DMAP assisted ligand exchange was applied as a novel phase transfer method for the synthesized Fe_3_O_4_-AuNPs. We report that oleylamine and oleic acid can be replaced by DMAP on the Fe_3_O_4_-AuNP surface to achieve solubility in water and, more specifically, increase the concentration of Fe_3_O_4_-AuNPs transferred. Size distribution and consistent shell formation of Fe_3_O_4_-AuNPs has a number of implications in a wide range of applications. Therefore, it is of importance to understand the effects of slight temperature deviations on the structure, size and consistency of the synthesized Fe_3_O_4_-AuNPs product. In addition, some bionanotechnology applications require a high concentration of water soluble Fe_3_O_4_-AuNPs for separation-based techniques, which was accomplished using DMAP as a phase transfer catalyst.

## Funding source

Natural Sciences and Engineering Research Council (NSERC) Discovery Grant 224070.
