# Supplementary material for: Synthesis, transfer, and characterization of core-shell gold-coated magnetic nanoparticles
Source: MethodsX. 2019 Feb 8;6:333–54. doi: 10.1016/j.mex.2019.02.006 (PMC6396083; doi:10.1016/j.mex.2019.02.006)
Supplement: Supplementary file 1 [file mmc1.pdf]

Supplementary material *and/or* Additional information:

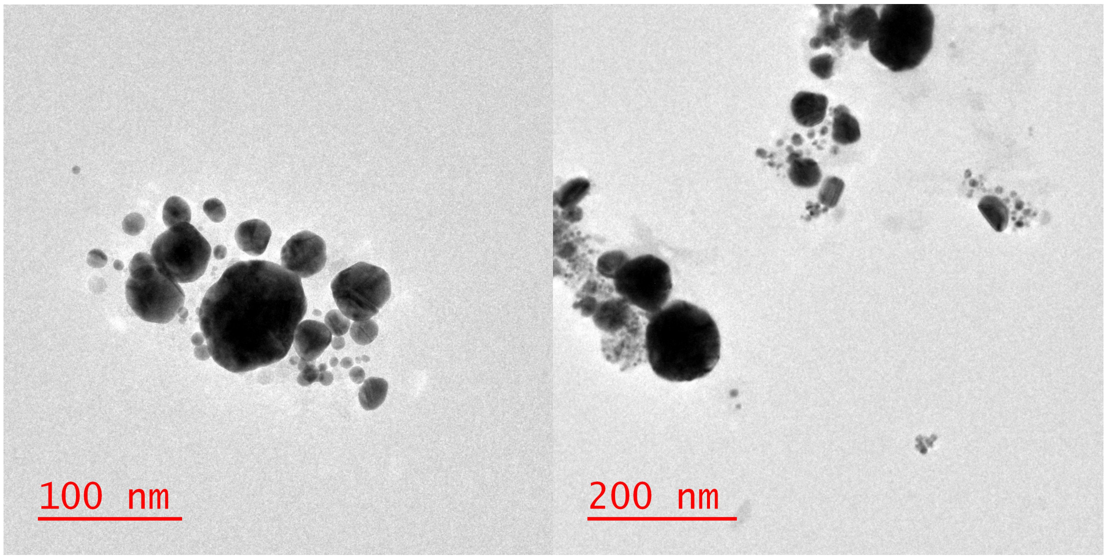

Fig. S3. HRTEM images of Fe_3O_4-AuNPs synthesized for 1.5 hours without an incremental increase in temperature (10℃/minute) (200 and 100 nm scale bars)

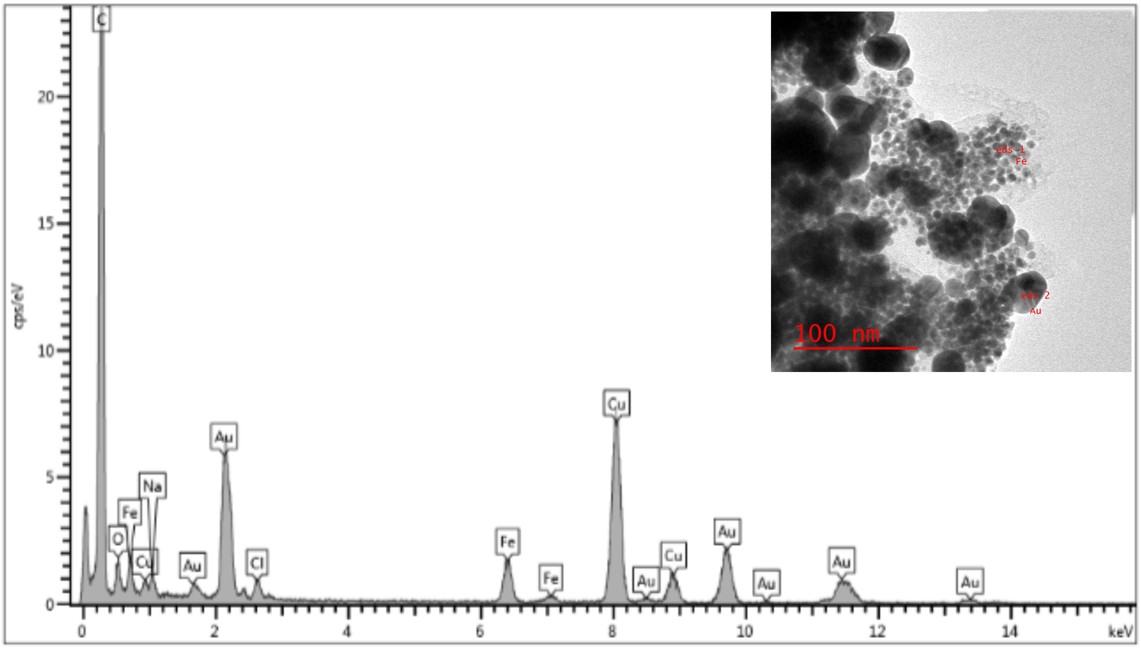

Fig. S4A. HRTEM image and corresponding EDS spectra of Fe_3O_4-AuNPs synthesized for 1.5 hours without an incremental increase in temperature (10℃/minute)

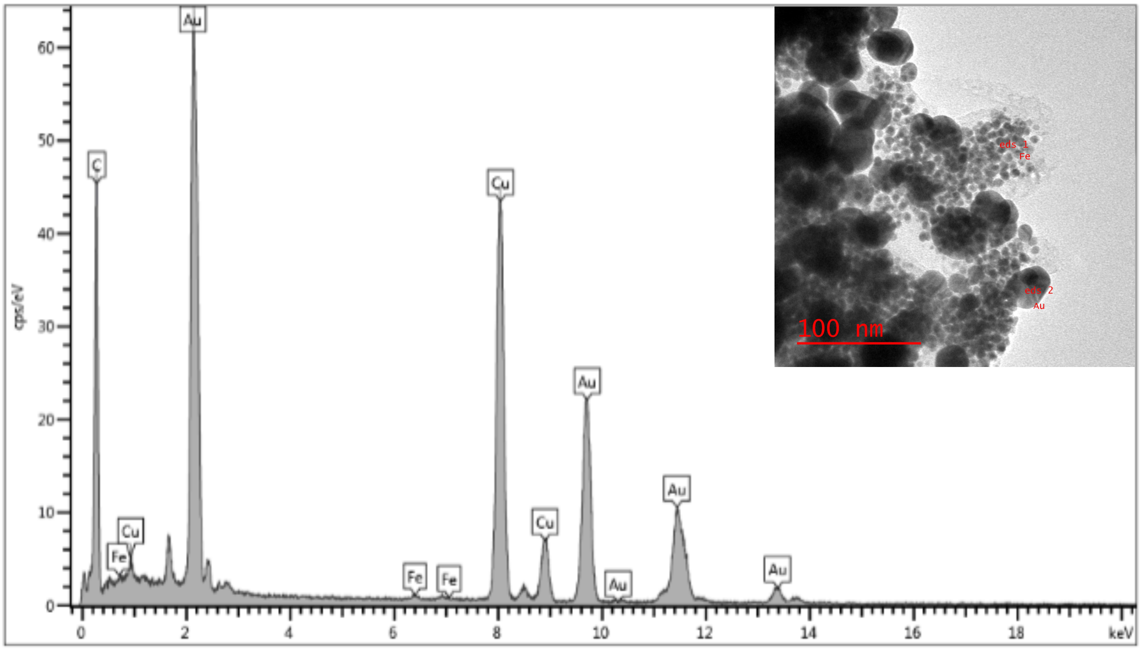

Fig. S4B. HRTEM image and corresponding EDS spectra of Fe_3O_4-AuNPs synthesized for 1.5 hours without an incremental increase in temperature (10℃/minute)

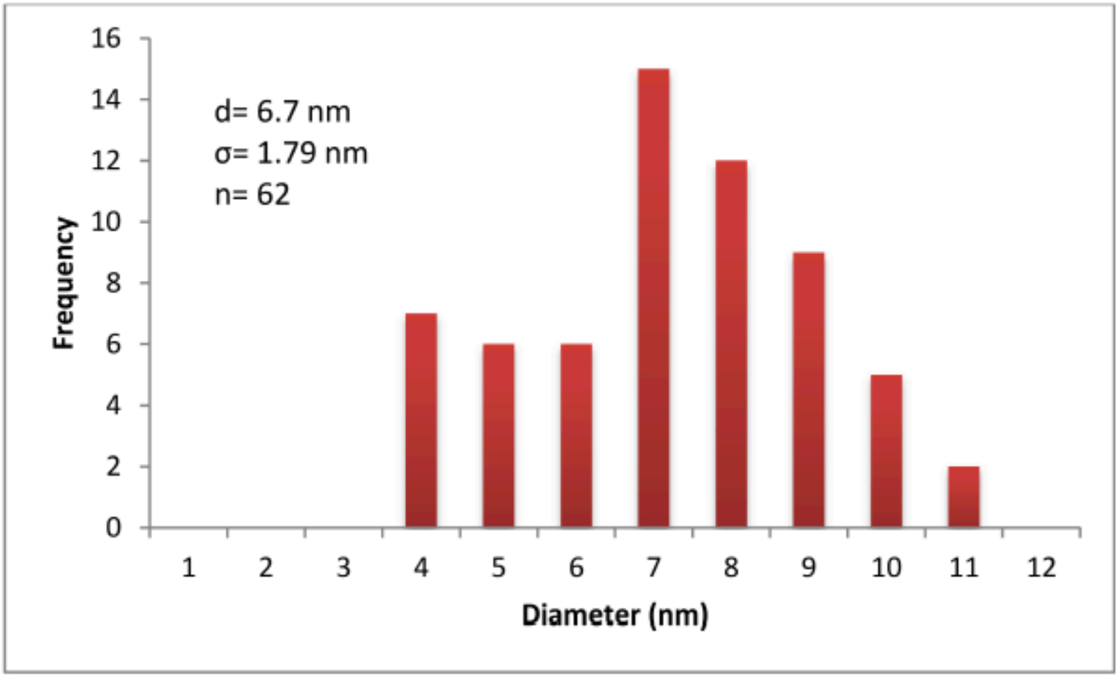

Fig. S5. Size distribution of Fe_3O_4-AuNPs synthesized for 1.5 hours without an incremental increase in temperature (10℃/minute)

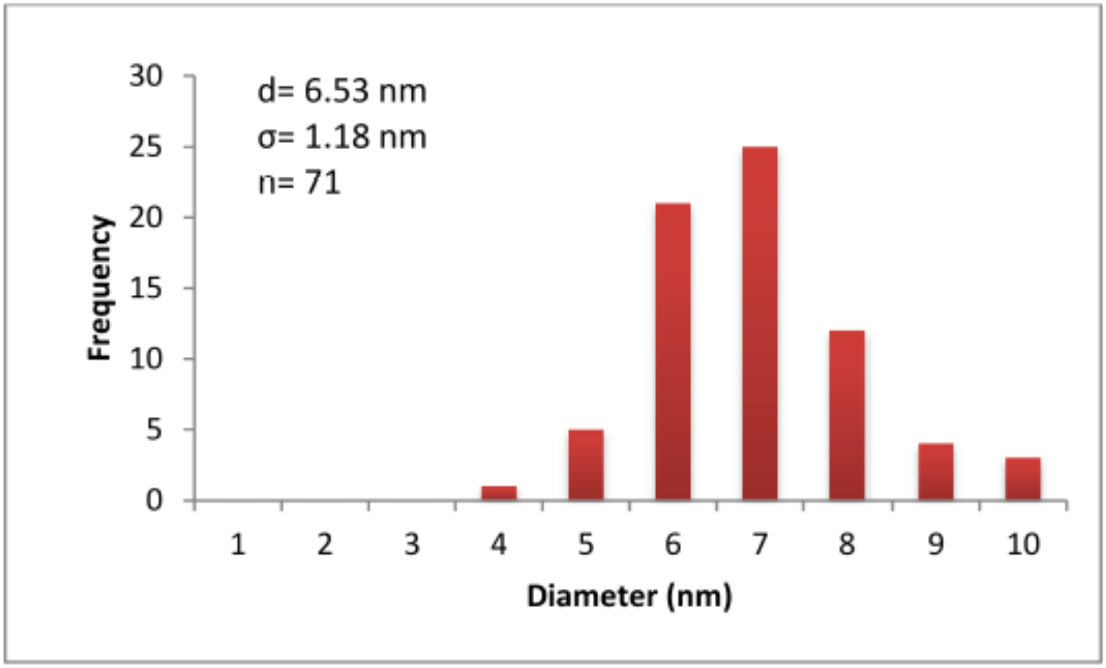

Fig. S6. Size distribution of Fe_3O_4-AuNPs synthesized for 3 hours with an incremental increase in temperature ($10\,℃$/minute)

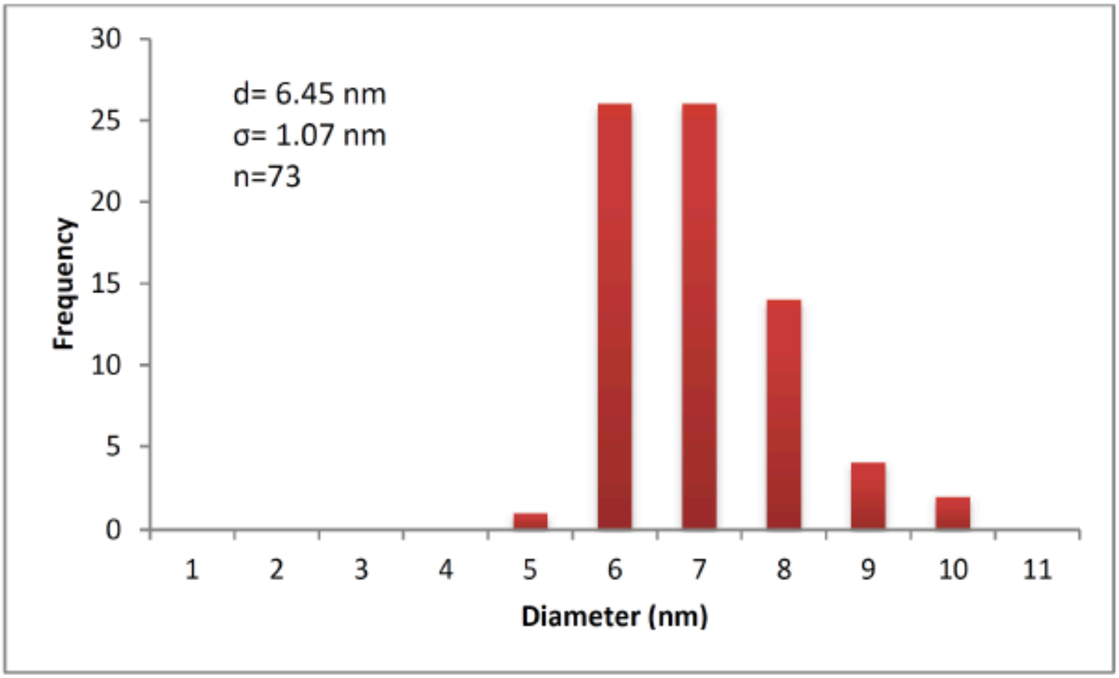

Fig. S7. Size distribution of DMAP transferred Fe_3O_4-AuNPs

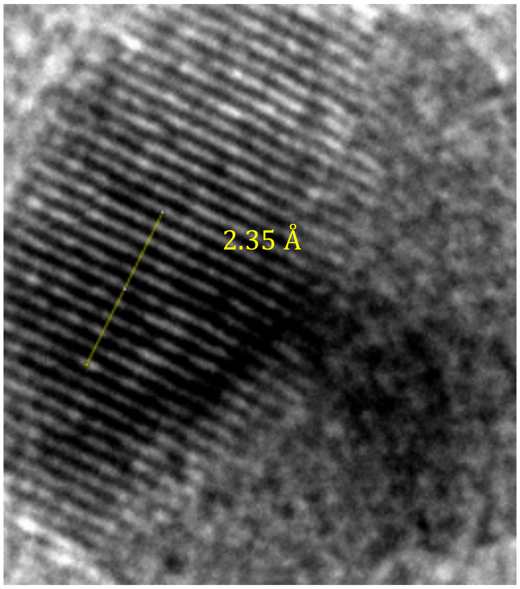

Fig. S8. HRTEM image of Fe_3O_4-AuNPs displaying the distance over an average of 10 lattice spacings

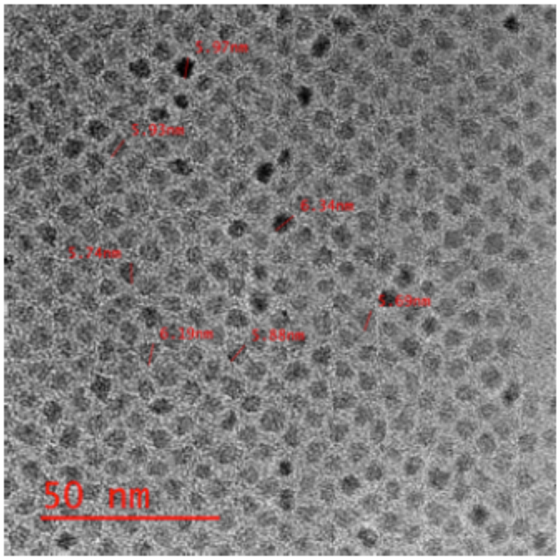

Fig. S9. HRTEM image displaying the average size distribution of Fe_3O_4 NPs with a 50 nm scale bar

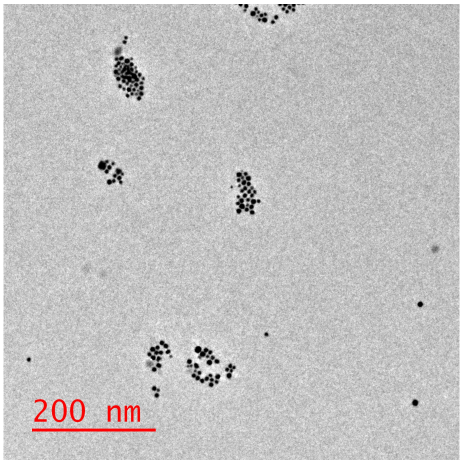

Fig. S10. HRTEM image of TMAOH transferred citrate-capped Fe_3O_4- AuNPs with a 200 nm scale bar

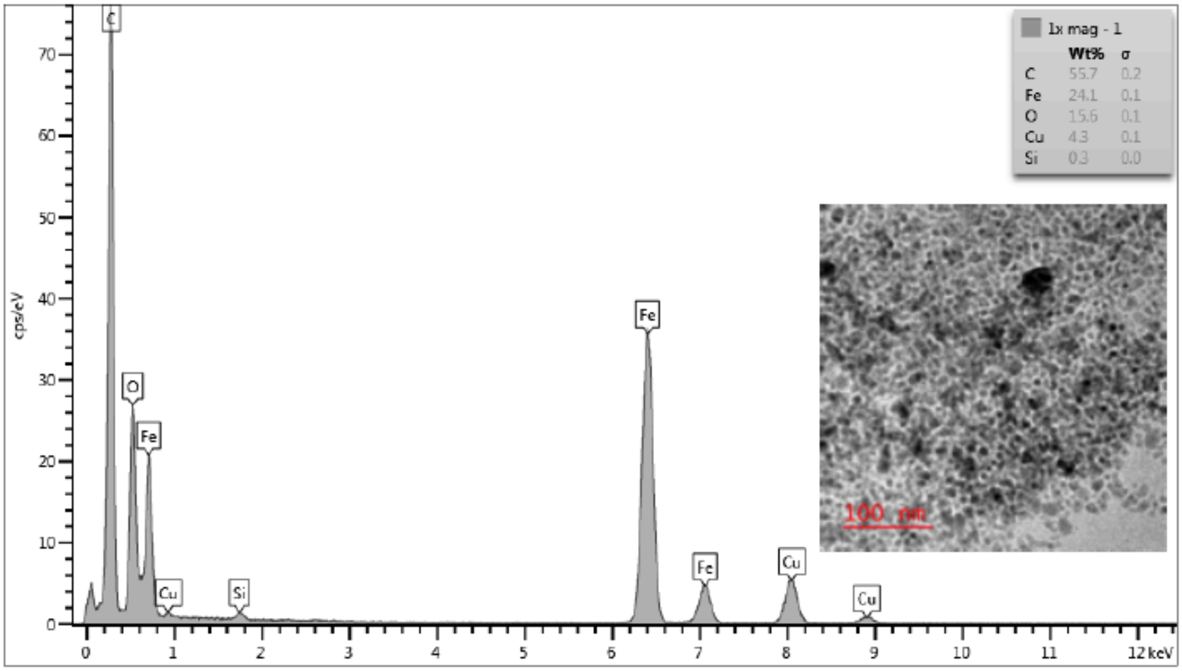

Fig. S11. TEM image with a 100 nm scale bar and associated EDS analysis of nanoparticles on the magnet after a mix of Fe3 O4 nanoparticles and AuNPs were separated by magnetic separation

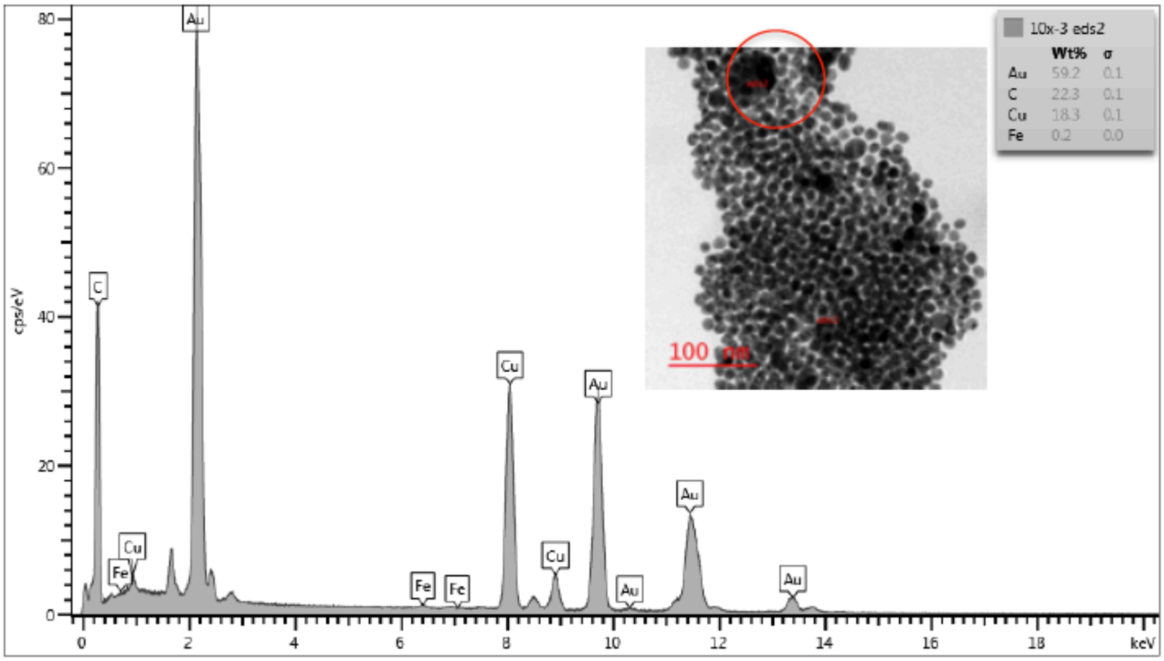

Fig. S11. TEM image with 100 nm scale bar and associated EDS analysis of nanoparticles in the supernatant after a mix of Fe3O4 nanoparticles and AuNPs were separated by magnetic separation
